# The relationship between self-care preparedness and quality of life in a 3-year-follow-up: a study in primary health care

**DOI:** 10.1093/fampra/cmae069

**Published:** 2024-12-03

**Authors:** Ulla Mikkonen, Nina Tusa, Sanna Sinikallio, Soili Marianne Lehto, Anu Ruusunen, Hannu Kautiainen, Pekka Mäntyselkä

**Affiliations:** Institute of Public Health and Clinical Nutrition, University of Eastern Finland, P.O. Box 1627, FI-70211 Kuopio, Finland; Wellbeing Services County of North Savo, Health Services, P.O. Box 1711, FI-70211 Kuopio, Finland; Institute of Public Health and Clinical Nutrition, University of Eastern Finland, P.O. Box 1627, FI-70211 Kuopio, Finland; Wellbeing Services County of North Savo, Educational services, P.O. Box 1711,FI- 70211 Kuopio, Finland; Independent Researcher, FI-73350 Haluna, Finland; Institute of Clinical Medicine, University of Oslo, P.O. Box 1171, Blindern, 0318 Oslo, Norway; R&D Department, Division of Mental Health Services, Akershus University Hospital, 1478 Lørenskog, Norway; Department of Psychiatry, University of Helsinki and Helsinki University Hospital, P.O. Box 22, FI-00014 Helsinki, Finland; Institute of Public Health and Clinical Nutrition, University of Eastern Finland, P.O. Box 1627, FI-70211 Kuopio, Finland; Wellbeing Services County of North Savo, Mental Health and Wellbeing, Kuopio University Hospital, P.O. Box 1711, FI-70211 Kuopio, Finland; School of Medicine, Institute for Mental and Physical Health and Clinical Translation (IMPACT), Food and Mood Centre, Deakin University, P.O. Box 281, Geelong, Victoria 3220, Australia; Folkhälsan Research Center, Topeliuksenkatu 20, 00250 Helsinki, Finland; Institute of Public Health and Clinical Nutrition, University of Eastern Finland, P.O. Box 1627, FI-70211 Kuopio, Finland; Wellbeing Services County of North Savo, Clinical Research and Trials Centre, Kuopio University Hospital, P.O. Box 1711, FI-70211 Kuopio, Finland

**Keywords:** primary health care, self-care, quality of life, patient-centred care, chronic disease, patient care planning

## Abstract

**Background:**

Measuring self-care preparedness may enable better support for patients in general practice.

**Objective:**

This study assessed the relationship between the self-care preparedness index (SCPI) and health-related quality of life (HRQoL) in a longitudinal analysis over 36 months.

**Methods:**

This was a secondary analysis of an intervention group of a randomized controlled trial. Participants were adults (*n* = 256) with hypertension, diabetes, or coronary artery disease in primary health care. The intervention group was included in the present study since they had answered SCPI as a part of the intervention. The relationship between SCPI and HRQoL (15D) and other outcomes were studied at baseline. The mean changes in SCPI and 15D were calculated from baseline up to 36 months. Regression-based analysis was used to study to what extent the baseline SCPI was associated with the change in SCPI and 15D and to what extent the change in SCPI was associated with the change in 15D.

**Results:**

At baseline, 15D, physical activity, self-rated health, life satisfaction, and patient activation measures had a positive linear relationship with SCPI. Body mass index and depressive symptoms had a negative linear relationship with SCPI. The longitudinal association between changes in SCPI and 15D was statistically significant and positive. The adjusted β was + 0.19 (95% confidence interval: 0.07 to 0.30, *P* = .002).

**Conclusion:**

Those patients who managed to increase their SCPI over the study period experienced an improvement in HRQoL.

**Key messages**

- Supporting self-care in primary health care is crucial, yet self-care preparedness varies among patients with chronic diseases.
- SCPI is a short generic tool for identifying self-care preparedness.
- SCPI correlated positively with health-related quality of life and its change over a 36-month follow-up period.

## Background

Self-care is a key component in the prevention and treatment of chronic conditions. Slightly different definitions for self-care exist, but there are some core similarities in the definitions of self-care and self-care across chronic conditions. Riegel *et al*. describe self-care as a process of maintaining health through practices that promote health and manage an illness. They define self-care maintenance, self-care monitoring, and self-care management as key practices of self-care. Maintenance refers to physical and emotional stability achieved by actions such as adherence to healthy behaviours or medications; monitoring refers to the process of observing oneself by different measurements, signs, and symptoms; and management refers to how an individual deals with these observations through daily decision-making [1]. Despite the importance of self-care, maintaining it may be challenging, especially under stressful circumstances.

Various personal factors such as motivation, self-efficacy, health literacy, physical, and psychological capability pose challenges to the maintenance of self-care [2–5]. Usually, the self-care of chronic diseases includes a commitment to health behaviour changes. When supporting individuals in their self-care, motivation, and capability as well as the opportunity to change should be considered [5]. Capability and motivation involve personal factors that have an impact on self-care, but the concept of opportunity refers to all factors outside the individual [5] such as community and system [6]. The continuity of care, e.g., is associated with a greater perceived control over one’s health, success of self-care, and health-related quality of life (HRQoL) [7]. In addition, without appropriate support, accessing health care or social-care services when carrying out self-care can be demanding [1, 6, 8]. Support from a social network and family are also important [8]. Thus, people have different opportunities to carry out self-care.

Measuring self-care preparedness may enable better support for patients in maintaining self-care. For patients, this is valuable since the success of self-care performance impacts health-related factors [9–11], HRQoL [12], and functional capability [3]. However, the need for support varies on an individual basis. Hence, measuring self-care preparedness as a part of care planning is reasonable and might aid in allocating healthcare resources better.

Previously, we have introduced a short self-care preparedness index (SCPI) for measuring self-care preparedness among patients with chronic conditions in general practice [13]. The aim of this study was to analyse the longitudinal associations between self-preparedness and HRQoL up to a 36-month follow-up.

## Methods

This was a secondary analysis of a randomized controlled trial that studied the effects of participatory patient care planning in general practice (Clinicaltrials.gov ID: NCT02992431) [14, 15]. The inclusion criteria for the trial were participants aged ≥ 18 years with hypertension, coronary artery disease, or diabetes. The trial took place in Finland, in the Siilinjärvi Municipal Health Centre during 2017 and 2021. Those patients who had an upcoming follow-up visit concerning their chronic condition management were recruited. Consenting participants were then randomly assigned to either the intervention or control groups. The intervention was a participatory patient care planning process in primary health care. Initially, the self-care form, including the SCPI, was mailed to the participants in the intervention group. The form explained what self-care means and included the assessments of health behaviours and willingness to change, as well as individualized goal setting. They were also asked to conduct self-care follow-up measurements at home (e.g. blood pressure and blood sugar) and to undergo blood tests for their chronic disease. After these, an individual care plan was completed at an appointment with a trained nurse and general practitioner (GP), and the patient received a written copy. The follow-ups related to chronic diseases were then carried out according to the individual care plan. The control treatment was usual care without the participatory patient care planning process. A thorough description of the study and the intervention protocol and flow diagram has been published previously [14, 15].

In the present study, we only used data from the intervention group, as they had completed the SCPI as a part of the intervention. A total of 301 participants were randomized to the intervention group. Of those, a total of 256 were included in the present analysis as they had completed the SCPI at baseline and had at least one 15D measurement at any follow-up point (12 or 36 months). There were 248 patients with a 15D measurement at 12 months and 256 patients at 36 months.

### Outcomes

The following outcomes were measured at baseline, 12 months and 36 months.

SCPI is a six-item measure for self-care preparedness and has been thoroughly described in a previous study [13]. SCPI was formed from the self-care questionnaire that the participants in the intervention group answered as a part of the intervention. The total score in SCPI varies from −5 to + 5 with higher scores reflecting higher self-care preparedness. In the present study, we divided the SCPI into tertiles in baseline analyses. An index value of −5 to 0 indicated low preparedness; 1 to 3 moderate preparedness; and 4 to 5 high preparedness. In longitudinal analyses, SCPI was used as a continuous measure.

The 15D is a valid measure for HRQoL. The 15D includes 15 dimensions (breathing, mental function, speech, vision, mobility, usual activities, vitality, hearing, eating, excretion, sleeping, distress, discomfort and symptoms, sexual activity, and depression). The scores range from 0 to 1 and higher scores indicate higher HRQoL [16]. The minimal clinically relevant change in 15D is estimated to be 0.015 points [17]. In Finnish adults, the age- and sex-weighted population-based mean for 15D is estimated to be 0.891 [18].

To measure depressive symptoms, we used the Beck Depression Inventory (BDI) where scores range from 0 to 63 and higher scores indicate more depressive symptoms [19].

The patient activation measure (PAM) was used to measure patient activation, which considers an individual’s knowledge, skills, and confidence in managing their overall health. In PAM, scores range from 0 to 100, and higher scores indicate higher levels of activation [20].

Self-rated health was measured with a single question by asking how participants would rate their health in general. The answer options were excellent, very good, good, fair, and poor [21]. The first three options were re-grouped for analysis as ‘good’.

Life satisfaction was measured with a single question by asking how satisfied the patients were with their life at that point [22]. The answer options were very satisfied, satisfied, somehow satisfied, unsatisfied, and very unsatisfied. The first three options were re-grouped as ‘satisfied’ for analysis.

Physical activity was evaluated with the FIT (Frequency Intensity Time) Index of Kasari, in which scores range from 1 to 100 with higher scores reflecting higher physical activity [23]. The FIT index considers the frequency and intensity of exercise (both scored on a scale of 1 to 5) and time spent on workout (scored on a scale of 1 to 4). The frequency ranges from ‘less than one time per month’ to ‘at least six times per week’. The intensity ranges from light walking to high-intensity training. The time ranges from ‘less than 10 min’ to ‘more than 30 min’.

Participants were also asked about their current smoking (yes/no, number of cigarettes per day), alcohol consumption (the first two questions of AUDIT-C, The Alcohol Use Disorders Identification Test-Concise) [24], pain, additional diseases, and socio-demographic factors (sex, age, education years, and living alone).

The trained study nurse measured weight (kg) and height (cm) and calculated body mass index (BMI) as weight (kg)/height (m^2^). Low-density lipoprotein cholesterol was measured at the laboratory after 12 h of fasting in accordance with the standard laboratory protocol of the Kuopio University Hospital.

### Statistical analysis

Data are presented as means with standard deviation (SD) or as counts (*n*) with percentages (%). The unadjusted hypothesis of linearity across categories of SCPI (tertiles) levels was tested using the Cochran–Armitage test, analysis of variance, or logistic models with appropriate contrast. Relationships between continuous SCPI at baseline and continuous SCPI change (SCPI at 36 months minus SCPI at baseline) were assessed by means of linear regression analysis [25]. Possible nonlinear relationships between continuous SCPI and 15D were assessed by using three-knot restricted cubic spline regression models. The models were adjusted for age, sex, and baseline depressive symptoms, BMI, SCPI at baseline, and 15D at baseline, when appropriate. The beta value is a measure of how strongly the predictor variable influences the criterion variable. The beta is measured in units of SD. Cohen’s standard for beta values above 0.10, 0.30, and 0.50 represents small, moderate, and large relationships, respectively. Regression models enabled the analyses of unbalanced datasets without imputation; therefore, all available data were analysed using the full analysis set (missing data were handled by using available-case analysis). Unadjusted and adjusted (partial) correlations were calculated with the Pearson method. The Stata 18.0 (StataCorp LP; College Station, Texas, USA) statistical package was used for the analysis.

## Results

Table 1 presents the characteristics of 256 participants according to tertiles of SCPI at baseline. There was a decreasing trend in the proportion of women from lower to higher tertiles (*P* = .037). Physical activity, self-rated health, life satisfaction, and PAM had a statistically significant positive linear relationship with SCPI. Conversely, BMI and depressive symptoms (BDI) had a statistically significant negative linear relationship with SCPI (Table 1).

**Table 1.** The characteristics of patients (*n* = 256) at baseline grouped according to tertiles of the SCPI in primary health care (2017–2018).

	Tertiles of SCPI			*P*-value^a^
	Low −5 to 0 *N* = 60	Moderate 1 to 3 *N* = 104	High 4 to 5 *N* = 92	
Women, *n* (%)	40 (67)	56 (54)	45 (49)	.037
Age, mean (SD)	69 (9)	67 (9)	69 (9)	.71
Number of education years, mean (SD)	9.6 (2.9)	10.2 (3.0)	10.3 (3.1)	.22
Living alone, *n* (%)	14 (23)	23 (22)	22 (24)	.90
Smoking, *n* (%)	7 (12)	12 (12)	9 (10)	.69
Alcohol consumption^b^	1.7 (1.4)	1.6 (1.4)	1.8 (1.5)	.80
Physical activity, mean (SD)	34 (20)	42 (19)	46 (18)	<.001
Chronic conditions, *n* (%)				.80
Hypertension	25 (42)	39 (38)	42 (46)	
Coronary artery disease	9 (15)	20 (19)	15 (16)	
Diabetes mellitus	26 (43)	45 (43)	35 (38)	
Additional diseases, *n* (%)				
Musculoskeletal disorders	40 (67)	46 (44)	49 (53)	.20
Psychiatric disorders	7 (12)	8 (8)	6 (7)	.28
Pulmonary diseases	5 (8)	16 (15)	13 (14)	.36
Dementia	1 (2)	2 (2)	0 (0)	.29
Number of chronic and additional diseases, mean (SD)	1.9 (0.7)	1.7 (0.7)	1.7 (0.7)	.28
Body mass index, kg/m^2^, mean (SD)	30.8 (5.2)	29.3 (5.4)	27.7 (4.3)	<.001
LDL cholesterol, mmol/l, mean (SD)	2.59 (0.95)	2.75 (0.97)	2.62 (0.99)	.97
Experiences of pain during the week, *n* (%)	43 (73)	65 (64)	61 (67)	.54
Rated their self-rated health as good, *n* (%)	20 (34)	60 (58)	65 (71)	<.001
Satisfied with life, *n* (%)	39 (67)	80 (78)	80 (88)	.002
Beck Depression Index score, mean (SD)	7.9 (5.7)	6.5 (4.7)	4.7 (4.5)	<.001
Patient activation (PAM), mean (SD)	66 (15)	70 (14)	72 (20)	.032

^a^ *P*-values for linearity.

^b^Alcohol consumption: the first two questions of AUDIT-C.

The mean (SD) 15D among the whole sample was 0.869 (0.096) at baseline. The total score of the 15D correlated positively, but low to moderate, with SCPI at baseline [*r* = 0.30 (95% CI: 0.19 to 0.41)] as did most of the dimensions of 15D (Table 2). The positive linear relationship appeared to be the most evident in the following dimensions: usual activities, discomfort and symptoms, depression, distress, vitality, and sexual activity.

**Table 2.** The relationships between the tertiles of SCPI and the 15D measurement for HRQoL at baseline among the sample of 256 primary health care patients (2017–2018).

15D dimensions	Tertiles of SCPI			*P*-value^a^
	Low −5 to 0 *N* = 60 Mean (SD)	Moderate 1 to 3 *N* = 104 Mean (SD)	High 4 to 5 *N* = 92 Mean (SD)	
Mobility	0.841 (0.177)	0.891 (0.174)	0.913 (0.148)	.011
Vision	0.913 (0.130)	0.928 (0.141)	0.954 (0.107)	.044
Hearing	0.875 (0.178)	0.910 (0.163)	0.921 (0.149)	.11
Breathing	0.818 (0.190)	0.812 (0.210)	0.868 (0.193)	.10
Sleeping	0.718 (0.210)	0.761 (0.193)	0.788 (0.207)	.041
Eating	0.982 (0.078)	0.993 (0.049)	1.000 (0.020)	.032
Speech	0.975 (0.083)	0.971 (0.088)	0.987 (0.061)	.30
Excretion	0.770 (0.238)	0.806 (0.230)	0.876 (0.178)	.002
Usual activities	0.835 (0.177)	0.883 (0.172)	0.926 (0.154)	.001
Mental function	0.853 (0.213)	0.859 (0.175)	0.918 (0.151)	.018
Discomfort and symptoms	0.626 (0.195)	0.711 (0.208)	0.723 (0.182)	.005
Depression	0.865 (0.161)	0.901 (0.140)	0.930 (0.114)	.004
Distress	0.852 (0.179)	0.881 (0.158)	0.928 (0.127)	.002
Vitality	0.787 (0.148)	0.808 (0.167)	0.871 (0.136)	<.001
Sexual activity	0.738 (0.277)	0.815 (0.284)	0.885 (0.199)	<.001

^a^ *P*-values for linearity.

**Figure 1.**
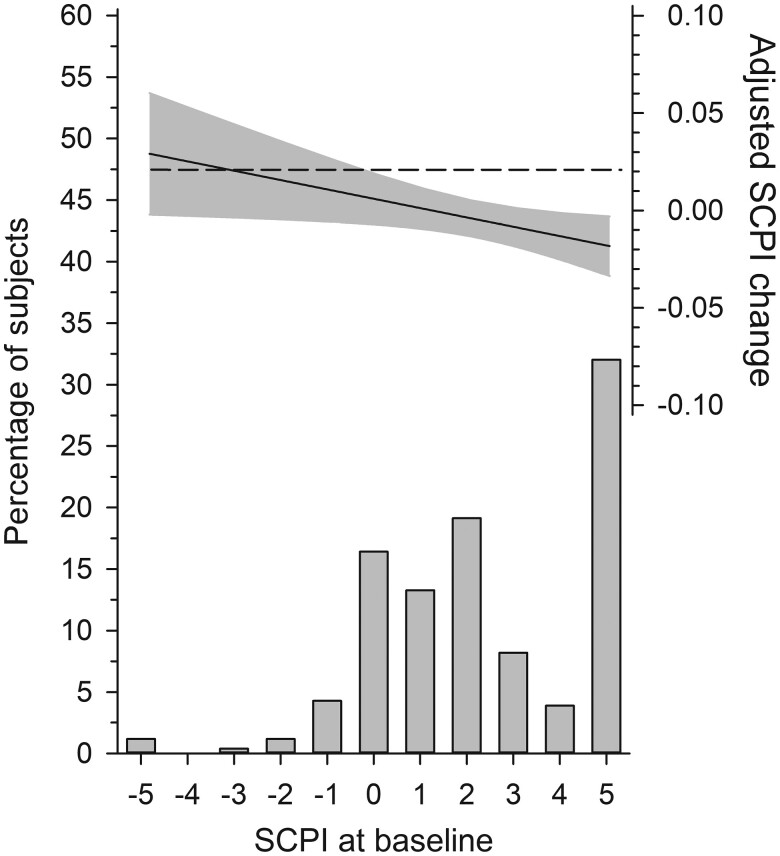
Adjusted SCPI change (SCPI at 36 months minus SCPI at baseline) as a function of the baseline SCPI. The curves were derived from a linear multivariate regression analysis. The dotted line shows the mean adjusted SCPI change. The grey area represents 95% CIs. The model was adjusted for age, sex, depressive symptoms, and the baseline SCPI.

Figure 1 presents how the baseline SCPI score was associated with a change in SCPI over the study period. The regression coefficient was β= −0.14 [95% confidence interval (CI): −0.27 to −0.01, *P* = .027] meaning that every unit increase in the baseline SCPI reduced the change of SCPI by 0.14 units at the follow-up. The whole sample’s mean change in SCPI was 0.20 (95% CI: −0.12 to 0.53, *P* = .23) from baseline to 36 months.

**Figure 2.**
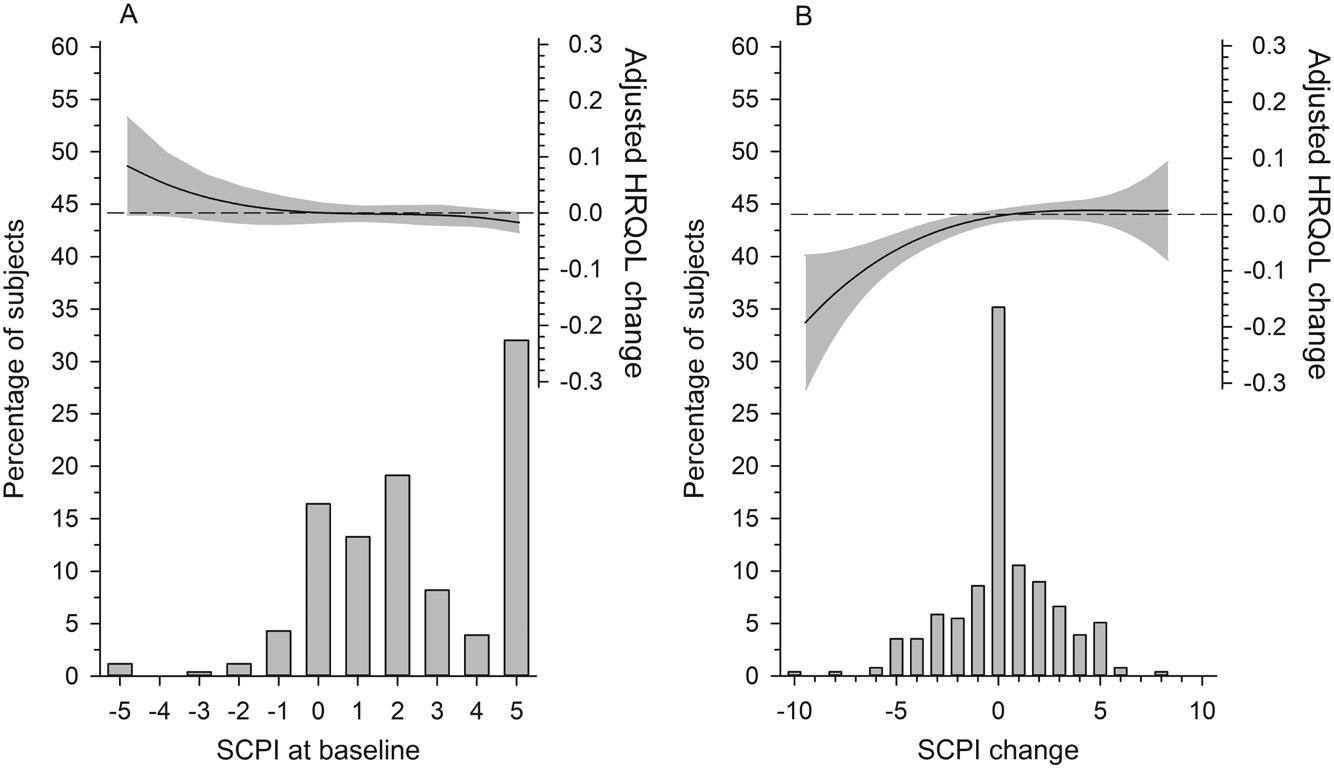
The figures present relationships between (A) baseline SCPI and change (B) as a function of health-related quality of life (15D) over the 36-month follow-up. The curves were derived from a three-knot restricted cubic spline regression model. The model was adjusted for age, sex, and baseline depressive symptoms, SCPI, BMI, and 15D. The change in SCPI and HRQoL was calculated as the 36-month measurement minus the baseline measurement. The grey area represents 95% CIs. The bars represent the percentage of the subjects.

**Figure 3.**
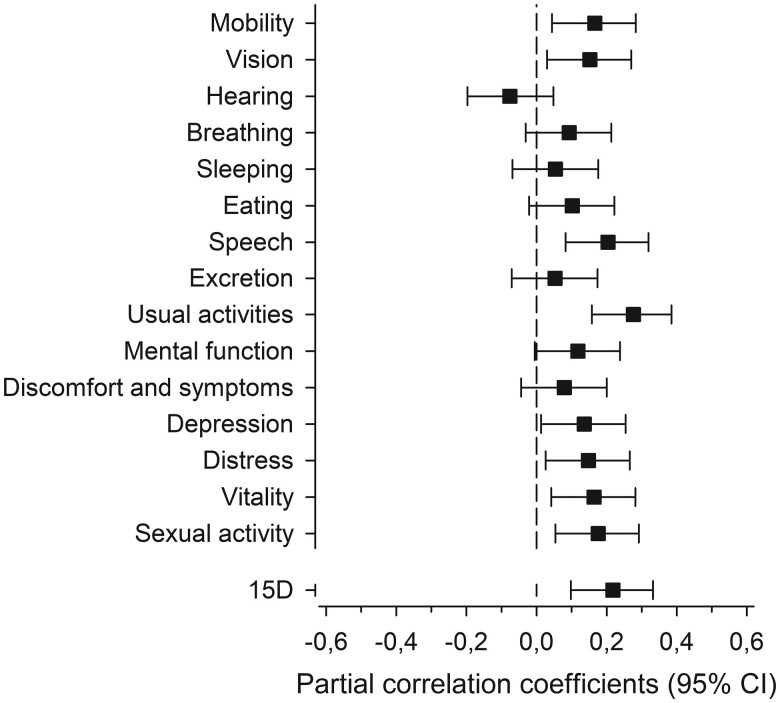
Partial correlations (adjusted for age, sex, and baseline BDI index, BMI, SCPI, and 15D dimensions) between the change in SCPI and the change in 15D measurement dimensions.

Picture A in Fig. 2 shows how the baseline SCPI was associated with a change in 15D over the study period. A lower SCPI at baseline was associated with a larger improvement in 15D. The right picture, B, in Fig. 2 presents the longitudinal association between the change in SCPI and the change in 15D from baseline to 36 months [β = + 0.19 (95% CI: 0.07 to 0.30), *P* = .002]. Figure 3 further depicts how most of the dimensions of the 15D correlated with the SCPI change. The mean change in the total score of 15D among the whole sample was not statistically significant from baseline to 36 months [−0.006 (95% CI: −0.015 to 0.003, *P* = .22)].

## Discussion

### Summary

This study provided information on SCPI as a measure for self-care preparedness in primary health care patients with chronic conditions. SCPI correlated positively and significantly with the 15D measure for HRQoL at baseline but also with its change in the follow-up. Hence, those patients who managed to increase their SCPI over the study period experienced improvement in their HRQoL. Conversely, when SCPI decreased, HRQoL decreased simultaneously. This is a meaningful finding from a clinical point of view since those patients with a lower SCPI score at baseline had also a greater need for improvements in clinical variables. Furthermore, they might also have the potential to improve their health, as an increase in SCPI had a positive association with HRQoL. This finding also provided support for the assumption that SCPI identifies patients who have varying needs for self-care support.

### Comparison with existing literature

As expected, the study participants, who suffered from chronic health conditions, reported a poorer HRQoL (the mean 15D was 0.869 at baseline) than the average adult population in Finland. In the Finnish population, the age- and sex-weighted value for 15D is estimated to be 0.891 on average [18]. HRQoL refers especially to those dimensions of the quality of life that have a connection with health; in particular, mental, physical, and social functioning, as well as symptoms and ailments [18]. Significantly, among the participants with the lowest SCPI values, most of the dimensions of the 15D measurement were remarkably low in comparison to the average population in Finland. In contrast, for those who reported higher self-care preparedness, HRQoL was close to or even above the Finnish population average in several dimensions. Hence, SCPI could be a valuable and practical tool to detect those patients who have the strongest need for support in self-care and the potential to increase their HRQoL. Along with the association between self-care and HRQoL [4, 10], HRQoL is also linked to healthcare utilization and costs [26, 27]. Thus, measuring SCPI and tailoring self-care support accordingly might help in targeting healthcare resources better.

A few studies have examined the longitudinal association between self-care and HRQoL. A study among participants with heart failure showed a longitudinal association between self-care and HRQoL over an 18-month period. They observed that psychological distress, mostly depression, affects that relationship [12]. We also found that patients with lower self-care preparedness experienced more depressive symptoms at baseline. Depression is well-known to be associated with self-care-related health behaviours and HRQoL. Hence, psychological well-being needs to be considered in treatment strategies to enhance self-care [28, 29].

However, the baseline depression score (BDI) was controlled for in the longitudinal analyses. We found the SCPI score to be an independent predictor for HRQoL. Thus, lower HRQoL among patients with lower self-care preparedness is not solely explained by having more depressive symptoms. Carrying out self-care is a challenging process that requires multiple individual capabilities to adapt to living with chronic conditions, manage daily challenges, and solve problems effectively. Self-efficacy is associated with HRQoL [30] and self-care success [31]; resilience and self-care across chronic conditions are associated with each other [32]; and motivation is a key component for health behaviour change [5].

However, the longitudinal association between self-care and HRQoL is not only explained by psychological factors. We observed that 15D dimensions, including those that did not reflect psychological well-being, were associated with the SCPI change. In addition, we found that participants with a higher SCPI score rated their health as higher, were more satisfied with their life, and reported a higher level of physical activity. Overall, successful self-care is likely to require comprehensive well-being.

The concept of functional capacity also seems to explain the relationship between self-care and HRQoL. Functional capacity is a concept affected by symptoms and ailments and the limitations they impose on performance and participation, but also by environmental factors and personal characteristics [33]. Functional capacity, especially among older people, is an indicator of HRQoL and overall health [3]. In addition, higher HRQoL is associated with a reduced risk of mortality [34, 35]. Furthermore, physical activity and physical health modify the relationship between self-care and HRQoL. In a systematic review, physical activity was consistently associated with better functional capacity, higher HRQoL, decreased mortality, and better mental well-being among older adults (> 60 years) [3]. In elderly individuals, physical health is proposed to be one key factor between psychological well-being and health, and the association between physical health and subjective well-being is bidirectional [36].

To support an individual in self-care, measuring self-care preparedness may be a useful tool. SCPI was able to identify the participants with detrimental health behaviours at baseline. Although intervention participants followed the same protocol, the baseline self-care assessment may have affected the individual care plan. We can assume that patients with low self-care preparedness at baseline received more intensive support in the subsequent months. This may have had influence (effect modifier) on their self-care preparedness and HRQoL. Lundqvist *et al*. examined which factors predict a benefit from a physical activity prescription in primary health care. They found that factors predicting a change in the level of physical activity in a 6-month follow-up were self-efficacy, preparedness to change, and confidence in readiness [37]. Another study suggested that there inevitably are different patient groups with varying levels of preparedness for a lifestyle change. Patients exhibiting pessimism and a sense of helplessness were more reluctant to adhere to healthy lifestyle behaviours [38]. Thus, the negative beliefs and attitudes of patients as well as their poor readiness create barriers to self-care.

### Strengths and limitations

The sample size was relatively small and drawn from a local area in Finland, which may limit the generalisability of the results. However, data were collected from a normal patient flow, which is a strength. Although all intervention participants followed the same protocol, including a GP appointment, there may have been variation in how the intervention was implemented in a real-life setting. We did not gather information on whether the GPs used SCPI to formulate the care plan. The study was implemented into the routine operations of the health centre, i.e. it was not conducted as a separate clinical trial. Some results, such as the home measurements, were solely used to plan the treatment of the patients and were not collected for research purposes. We did not collect data on missing home measurements.

We had patient-reported outcomes complemented by clinical outcomes, which provided subjective and objective outcomes. The strength of the study is its clinical relevance. The study contributes to the knowledge on how to plan more individualized care and support patients better in daily decision-making. It is assumable that self-care questionnaire has enhanced the possibility to target support to those who need it most. Therefore, it is important to examine in the future whether SCPI can aid the targeting of self-care support in primary health care.

### Implications for research and/or practice

SCPI’s properties should be studied in different populations. Future studies should also examine whether patients with a low SCPI score would benefit from individualized self-care support.

SCPI could be used as an indicative index, keeping in mind that participants with lower SCPI have the potential to benefit and change their health behaviour the most. The patient and the health care provider should consider which areas of self-care the patient needs support. This study provides further knowledge of this tool for the purpose of aiding healthcare professionals in screening self-care preparedness in primary healthcare.

## Conclusions

At baseline, SCPI was associated with the variables that reflect health behaviour as expected. A lower SCPI at baseline was associated with a larger improvement in HRQoL over the study period. Those patients who managed to increase their SCPI also improved HRQoL.

## Data Availability

The data are available from NT upon reasonable request. Due to the protection of individual privacy, the data are not publicly available.
